# Occurrence, Temporal Variability, and Loads of Poly(vinyl chloride) in the Rhine and Moselle

**DOI:** 10.1021/acs.est.5c03236

**Published:** 2025-07-07

**Authors:** Jan Kamp, Georg Dierkes, David Range, Thomas Hoffmann, Thomas A. Ternes

**Affiliations:** † Federal Institute of Hydrology, Am Mainzer Tor 1, Koblenz 56068, Germany; ‡ University of Koblenz, Universitätsstraße 1, Koblenz 56070, Germany

**Keywords:** microplastics, suspended matter, time-resolved analysis, temporal behavior, annual loads, combustion ion chromatography

## Abstract

There is an urgent need to improve the knowledge about occurrence and behavior of microplastics in the aquatic environment. The aim of this study was to determine the temporal variability and spatial distribution of poly­(vinyl chloride) (PVC) in the larger German rivers Rhine and Moselle. Monitoring campaigns using a continuous flow centrifuge (sampling of suspended matter in a particle size range of 1 mm–1 μm) revealed a strong dependence of PVC concentrations of up to 3.3 μg/L on the discharge. It could be shown that PVC microplastic concentrations have strong correlations with suspended matter (correlation coefficients >0.94 with *p*-values <0.05). PVC microplastics and suspended matter were thus evidenced to show an identical temporal behavior in the Rhine River and Moselle. Furthermore, our results indicate that the pollution with PVC microplastic is mainly originated from diffuse rather than point sources. Elevated PVC loads were mainly observed during floods. Our study confirmed that approximately 80% of the PVC loads can be exclusively attributed to these high discharge events. Calculated annual PVC loads (2019–2022) ranged from 2–17 t/a (Moselle) to 10–38 t/a (Rhine). In addition, long-term trend analysis of loads (2006–2022; particle size range: <2 mm) at the Rhine for Weil, Iffezheim, Koblenz, and Bimmen showed significant decreasing trends (ranging from 0.41 to 1.72 t/a) for all these sites with an overall decrease ranging between 38 and 58%.

## Graphical abstract

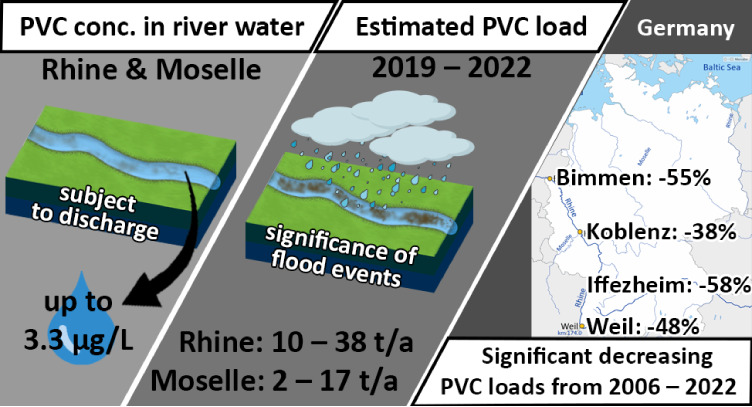

## 1 Introduction

Plastic, a diverse class of synthetic polymers, has become omnipresent in modern life due to its versatility, durability, and cost-effectiveness. The most commonly produced plastics, with annual production volumes of up to 75.6 Mt, are polyethylene (PE), polypropylene (PP), poly­(vinyl chloride) (PVC), polystyrene (PS), polyethylene terephthalate (PET), polycarbonate (PC), and acrylonitrile butadiene styrene (ABS).^1^ Each plastic type serves specific purposes, from packaging and construction to automotive and medical applications.^1^ While plastic applications are essential for industrial developments, concerns about their environmental impacts and waste management solutions remain a key challenge. PVC is the third most commonly used synthetic polymer with multiple applications. In 2022, PVC volume reached a share of 9.1% in Europe (5.3 Mt) and of 12.7% of total global plastic production (50.8 Mt).^1^ Today, PVC is mainly used in building and construction, but also in packaging, electronics, the mobility sector, household applications, and many other fields.^1–3^ Moreover, PVC products are manufactured with an additive content of up to 50%.^4,5^ These additives encompass a range of substances, including heat stabilizers, plasticizers, processing aids, lubricants, and flame retardants.^4,5^ The potential environmental impact of PVC and its additives underscores the necessity for comprehensive and systematic elucidation of their occurrence and behavior in aquatic ecosystems.^6–9^

Plastics can make their way into the environment, especially into river systems, in the form of both macroplastics (particle size ≥ 5 mm) and microplastics (MP; particle size 1 μm–5 mm). MP are generated through various processes, including fragmentation of larger particles, abrasion of plastic-based materials, and the breakdown of plastic containing waste in landfills. One significant source is the weathering of plastic products in the environment, where they are exposed to sunlight, wind, and temperature fluctuations, which can lead to the mechanical breakdown of the materials.^10–12^ In addition, plastic-based textiles and other consumer goods release MP during use and washing, contributing to the dispersion of MP into aquatic ecosystems.^13–15^ Scientific studies have documented the widespread distribution of PVC MP in environmental compartments. Lassen et al.^3^ found PVC and other polymer particles in marine sediments, indicating the transport of these MP from inland to marine waters. Since wastewater treatment plants (WWTPs) have varying removal rates of MP ranging from 55–98% (depending on the processes used and regional conditions), emissions into riverine systems cannot completely be avoided. Thus, WWTPs are nonnegligible sources of MP, especially during heavy rain events leading to an elevated MP transport.^16,17^ A similar behavior has been observed in the context of combined sewer overflows (CSOs), which have been identified as notable sources of MP during periods of substantial rainfall.^18–20^ In addition to the aforementioned sources, industrial discharges and aquacultures have been identified as potential contributors to MP pollution. As a consequence, these sources contribute to the MP concentrations and associated MP loads which can be determined in inland waters and the marine area.^21–26^ The transport and fate of MP in rivers is subjected to a variety of interacting factors. Their sedimentation rate is a function of multiple characteristics including density, size, and agglomeration/interaction with organic matter or other particles.^27,28^ The denser a particle, the more likely it is to settle rapidly in water bodies.^29–31^ Conversely, lower density MP remain suspended for longer periods, although turbulence and hydrodynamic conditions may speed up their eventual deposition on the riverbed.^32,33^

In the literature, systematic studies dealing with the spatial and temporal distribution of MP in river systems mainly used optical methods for determining MP concentrations and loads such as microscopy, Raman spectroscopy or Fourier-transform infrared spectroscopy.^34–38^ However, these state-of-the-art methods are subjected to particle size limitations and cannot detect particles smaller than 20 μm.^39–42^ Disregarding smaller particles entails underestimations of calculated MP loads. In addition, MP load calculation is often based on the detection of particles per time, whereby the actual mass information is lost. An alternative analytical method is mass-based analysis using thermoanalytical methods, such as thermogravimetric analysis or pyrolysis coupled with gas chromatography/mass spectrometry (GC-MS).^43–51^ However, there is still a lack of studies calculating mass-based MP loads over longer time periods.^52^ One reason is that validated analytical methods for environmental samples are often unavailable. Unfortunately, mass-based methods used for MP analysis suffer from matrix effects resulting in under- and overdetermination of MP concentrations: Specific quantification markers lead to deviating concentrations in different matrices. Furthermore, it is known that the use of unspecific marker compounds for MP determination with pyrolysis GC-MS, e.g., naphthalene or benzene for PVC quantification, can lead to false-positive results and overestimations.^10,34,47,53–58^ In this context, our study focuses specifically on PVC microplastics, as this polymer is not only among the most widely produced plastics worldwide but also contains a high proportion of ecotoxicologically relevant additives. Moreover, the applied sampling technique using a continuous flow centrifuge (CFC) is particularly well-suited for the collection of PVC particles, as the high density of PVC (approximately 1.4 g/cm^3^) enables efficient retention and separation from the water phase.^59,60^ In addition, our previously developed method for quantification via combustion ion chromatography (C-IC) combined with pressurized liquid extraction (PLE) offers a selective and robust method for detecting PVC.^61^ In contrast, other polymers such as PE, PP, PS, or PET cannot be analyzed with this method due to the lack of distinct, selective marker compounds for the C-IC. This highlights the urgent need for refined analytical methods to enable accurate determinations of mass-based MP concentrations. In summary, systematic studies into the temporal behavior and transport of PVC are essential to determine the exposure of rivers and streams, to assess insights into the ecotoxicological relevance of MP and to initiate action toward reducing MP sources that are at the origin of the determined loads.^7^ Thus, the aim of this study was to elucidate the spatial and temporal distribution, and loads of PVC in the German rivers Rhine and Moselle, using a recently developed combustion ion chromatography method reported by Kamp et al.^61^ to quantify PVC MP in environmental samples such as suspended matter (SM). We applied this method to samples collected as a part of a long-term water quality monitoring program with a view to better understanding the dynamics of PVC pollution in larger rivers.

## 2 Materials and Methods

### 2.1 PVC Concentration in Riverine Suspended Matter

In order to investigate the temporal variability of PVC concentrations in rivers, SM samples were collected every 2 weeks from the Rhine (kilometer 590.4; Koblenz, Rhineland-Palatinate, Germany; Figure 1) and every 4 weeks from the Moselle (kilometer 2.0; Koblenz; Figure 1) over a total of four years (2019–2022). All samples were obtained using a CFC (Carl Padberg Z61 G, Padberg, Lahr, Germany). CFC sampling was carried out in the Rhine at approximately 5 m distance from the left bank and in the Moselle at approximately 5 m distance from the right bank with a sampling depth of 0.5 m for both sampling sites. The mean sampling volumes were 6 ± 1 m^3^ (Rhine) and 9 ± 5 m^3^ (Moselle) with mean sampling times of 7 ± 1 h and 9 ± 5 h for the Rhine and Moselle, respectively (see Tables S.2–5 for individual sample details).

**Figure 1:**
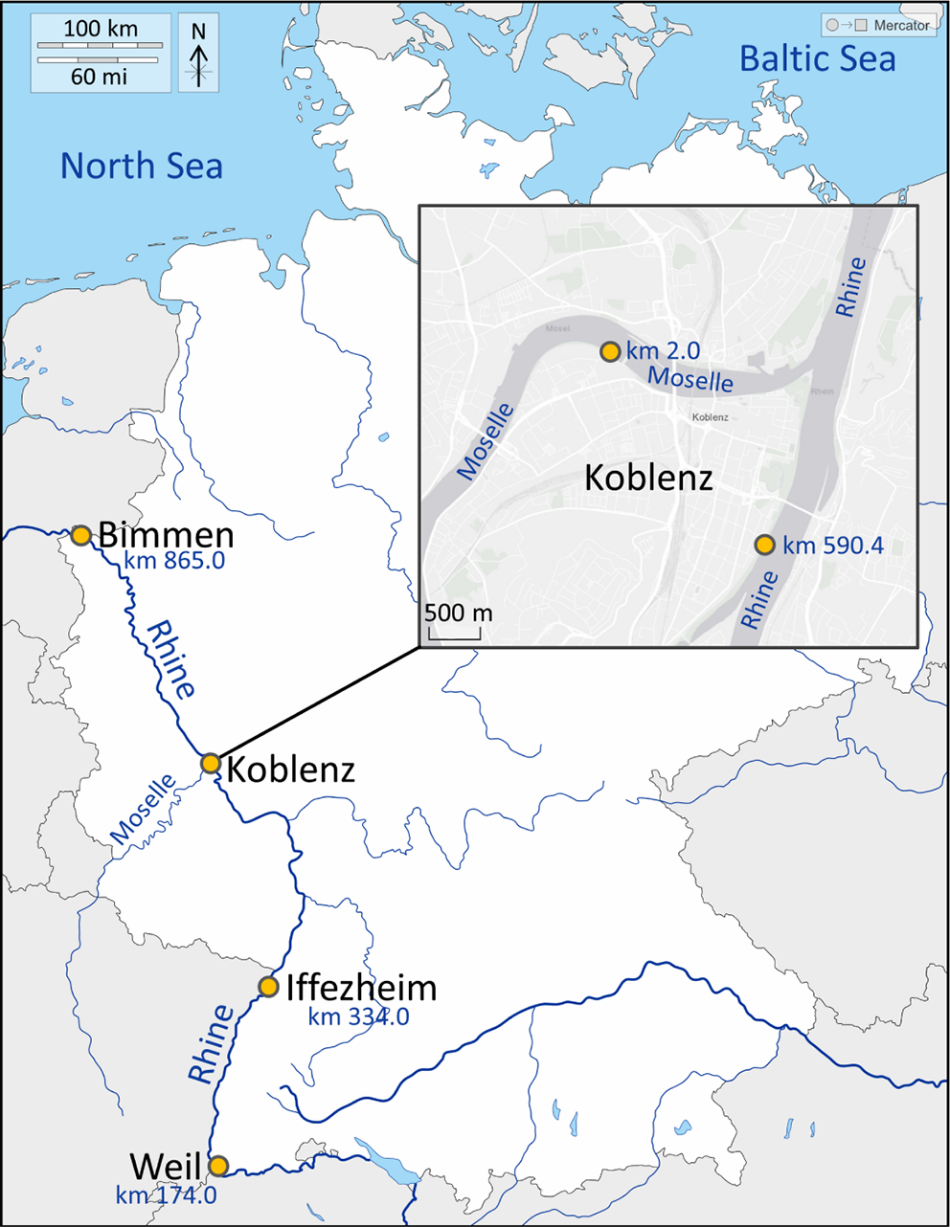
Map of Germany with all monitoring sites (marked with yellow dots) and corresponding river kilometers. Created by modifying a base map of Germany originating from d-maps.com (used with permission referring to the terms and conditions of https://d-maps.com; original map: https://dmaps.com/carte.php?num_car=2012&lang=en), accessed on 05.05.2025).

Since there is no generally accepted method for calculating a representative plastic load of rivers, the calculation used in this study is based on internationally established methods for determining SM loads or particulate nutrient and pollutant loads.^62^ Accordingly, the instantaneous PVC load *L*_*i*_ (μg/s) at the time of sampling (indicated by the index *i*) was calculated by the product of PVC concentration in SM *c*_*i*_ (μg/mg), SM concentration *s*_*i*_ (mg/m^3^) and discharge *Q*_*i*_ (m^3^/s):

1 $$L_{i}=c_{i}\cdot s_{i}\cdot Q_{i}$$

Finally, annual loads (t/a) were calculated for both locations for all four years by summing up the interval loads, which were determined based on the calculated instantaneous PVC concentrations/loads considering the respective time intervals between individual samplings. Data on river discharge and SM concentration is derived from the monitoring conducted by the Federal Waterways and Shipping Administration. SM concentrations were derived based on 15 min turbidity measurements using a Solitax TS-line sensor (Hach-Lange, Düsseldorf, Germany). This sensor is corrected by means of regular calibration using filtered water samples.^63^ Discharge data is generated from water level measurements at the closest river gauge of each sampling site.

In addition to instantaneous sampling of the Moselle and Rhine in Koblenz, a trend analysis of PVC loads was performed for a period of 17 years (2006–2022) at Weil (kilometer 174.0; Baden-Württemberg, Germany), Iffezheim (kilometer 334.0; Baden-Württemberg), Koblenz (kilometer 590.4) and Bimmen (kilometer 865.0; North Rhine-Westphalia, Germany) on the Rhine River (Figure 1). Therefore, annual composite samples from 12 monthly samples (January–December) were mainly analyzed, in most cases every two years (exceptions: additional 2019 and 2021 analysis for Koblenz and annual analysis from 2006 to 2015 for Iffezheim). All annual composite samples were provided by the German Environmental Specimen Bank (Schmallenberg, Germany). Monthly samples were collected using sedimentation boxes that were permanently installed and positioned at a depth of 0.5 to 2 m below the water surface. The collected SM was immediately preserved by shock freezing in liquid nitrogen. At the end of the year, the monthly samples were combined into an annual composite sample, which were then freeze-dried and homogenized (further details on sampling and sample preparation are described in Schulze et al.^64^). Average annual PVC concentrations were multiplied with annual SM loads from the SM monitoring network operated by the Federal Waterways and Shipping Agency and the Federal Institute of Hydrology.^65^ Time trends of the Rhine were analyzed for Weil, Iffezheim, Koblenz, and Bimmen using a software tool proposed by the German Environment Agency (UBA; LOESS-Trend, Version 1.1, based on Microsoft Excel). This tool uses a locally weighted scatterplot smoothing (LOESS) to fit a smooth curve through a scatterplot of annual contaminant levels and then tests the significance of linear and nonlinear trend components by means of an analysis of variance (ANOVA) following the approach described by Fryer and Nicholson.^66^

### 2.2 Sample Preparation and Extraction

All CFC samples were freeze-dried and homogenized using a planetary mill (Fritsch, Idar-Oberstein, Germany). All CFC and trend analysis samples were subjected to PLE according to Dierkes et al.^43^: The extraction involved cleaning with methanol (100 °C, 100 bar) and extracting with tetrahydrofuran (THF; 185 °C, 100 bar), with PVC being adsorbed onto 200 mg of silica gel as collection medium for analysis and calibration. For calibration, additive-free PVC (PyroPowders.de, Erfurt, Germany) in a concentration range of 5.0–0.01 mg/g was extracted and analyzed. After extraction, THF was evaporated using a TurboVap evaporation system (Biotage, Sweden) and remaining silica gel extracts were then ground and homogenized in a mortar. For the extraction of heated sea sand spiked with 1.0 mg/g PVC a recovery of 91.9 ± 5.5% (*n* = 8) was determined.^61^ A limit of quantification with a 95% confidence interval of 7.3 μg/g was calculated (based on mean extraction blanks; *n* = 45). Further details and calibration curve are described in Section S1.

### 2.3 Quantification via Combustion Ion Chromatography

C-IC (combustion ion chromatography) measurements were performed in accordance with Kamp et al.^61^ by combusting 20 mg of each silica gel extract at 1000 °C, followed by chloride detection using ion chromatography. PVC content was determined through the chloride concentration in the absorbent solution. Further details are described in Section S2.

### 2.4 Quality Assurance and Control

Only consumables and laboratory equipment were used that did not contain any PVC and had not been in contact with PVC. To prevent PVC contaminations, it was also made sure that the laboratory staff only wore cotton and safety clothing that were free of any plastics. All glassware and laboratory equipment used were cleaned with ethanol (HPLC grade; Merck, Darmstadt) before each step. All extraction cells were precleaned with Milli-Q water (Merck) and acetone (pico grade; LGC Standards, Wesel) and were heated at 100 °C for 1 h. All vessels were also cleaned with acetone. The glass fiber filters were furnaced at 450 °C for 2 h in a muffle furnace. In addition, silica gel and sea sand were heated at 700 °C for 2 h. Ceramic boats used were heated at 1000 °C for 1 h. Furthermore, device blank values and control standards were regularly extracted/measured to identify possible secondary contamination and to confirm the cleanliness of the extraction system (extraction cells filled with sea sand) and the C-IC setup (empty ceramic boats). The mean extraction blank value was 3.1 ± 1.6 μg/g (mean ± confidence interval; *n* = 45).

## 3 Results and Discussion

### 3.1 Spatial and Temporal Analysis of PVC Concentrations in Rivers

#### 3.1.1 Temporal Trends of PVC Concentrations in Suspended Matter

Mean PVC concentrations in SM of the rivers Rhine and Moselle in Koblenz between 2019 and 2022 (Figures S.4, 5 and Tables S.2–5) exhibited a low variance without significant differences (Levene test: *p* = 0.052 (Rhine), *p* = 0.859 (Moselle)) between the four years investigated (Rhine: 20 ± 3 μg/g (2019), 19 ± 2 μg/g (2020), 22 ± 3 μg/g (2021), 22 ± 3 μg/g (2022); Moselle: 26 ± 4 μg/g (2019), 21 ± 4 μg/g (2020), 25 ± 4 μg/g (2021), 24 ± 4 μg/g (2022); yearly mean values are reported with standard deviations arising from the measurement uncertainty of the C-IC method.^61^ In addition, the PVC MP concentration (μg/L) was calculated using the absolute PVC mass and the sampled volume (see Tables S.2–5). The results showed several peaks with elevated PVC concentrations of up to 3.3 μg/L (Figures S.2 and 3). These samples coincided with events marked by high discharges and SM loads, indicating a high significant correlation between PVC and SM concentrations for both rivers (Figure 2), with Spearman rank correlation coefficients >0.94 (*p*-values <0.05; Table S.6).

**Figure 2:**
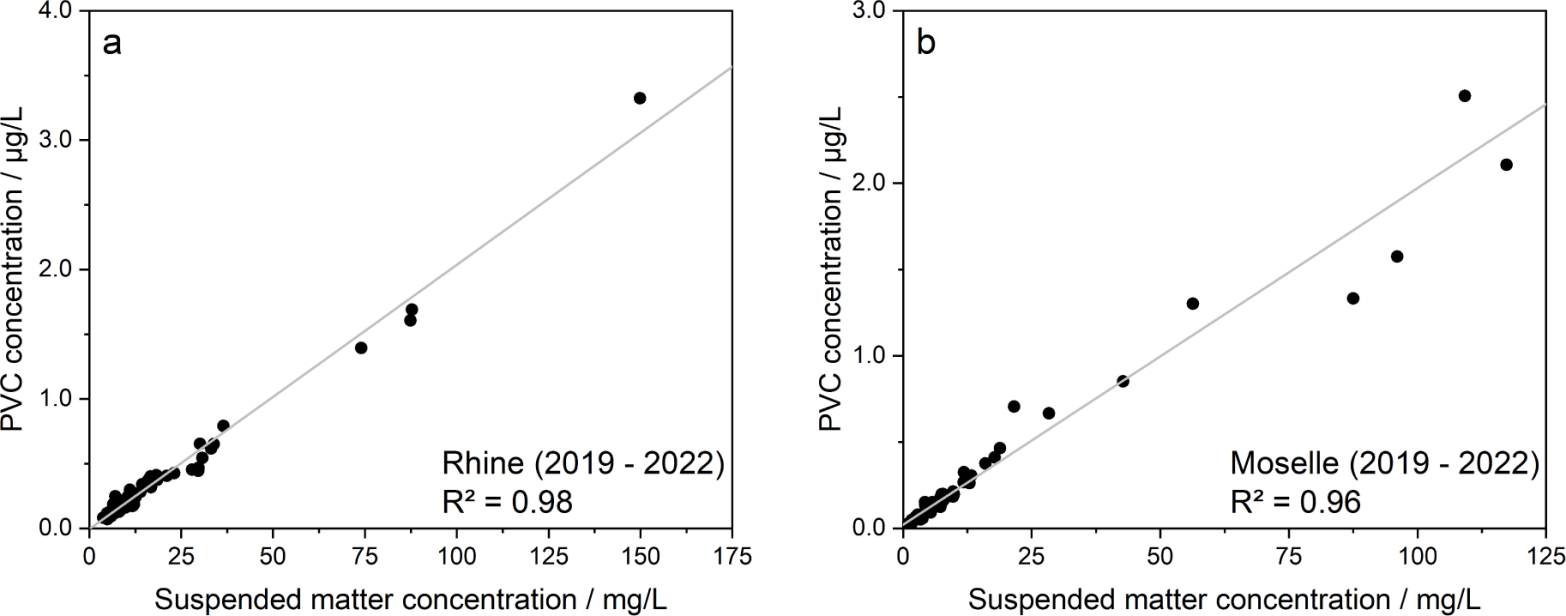
Plots of the PVC concentration via the SM concentration for the Rhine (a) and the Moselle (b) for all years considered (2019–2022). Gray lines represent the linear fit. Both sampling sites show a high linear correlation.

Figure 3a,b illustrates that the concentrations of SM increased with discharge, as it has been frequently observed for rivers in Germany (e.g., Rhine, Weser, Ems, Elbe) and other countries (e.g., Yangtze (China), Trinity River (California), Santa Ana River (California)).^67,68^ The low variability of PVC concentrations with respect to SM indicate the comparable relationships between PVC MP and the discharge of Rhine and Moselle (Figure 3c,d). At both rivers a slowly increasing gradient with discharge volumes of up to approximately 2000 m^3^/s for the Rhine and up to approximately 400 m^3^/s for the Moselle was identified, while a distinct break with a sudden increase could be observed for discharges >2000 m^3^/s and >400 m^3^/s, respectively (logarithmic axis scaling of Figure 3 shown in Figure S.6). This indicates that a discharge threshold for the transport of both SM and PVC applies to both rivers. Similarly distinct break points in sediment rating curves were found by Hoffmann et al.^68^ at many gauging stations on various German river systems: Break points in sediment rating curves occur due to varying sediment sources and transport processes depending on discharge rates. At low flows and especially during spring and summer months, the percentage of organic SM increased, sourced from in-stream production such as phytoplankton, with limited sediment mobilization.^68^ However, the majority of the total SM still consists of inorganic components.^68^ At high flows, increased discharge means that distal sediment sources, such as hillslopes and floodplains, become more closely connected. This results in a surge of mineral-rich sediment, involving distinct scaling behaviors.^68^

**Figure 3:**
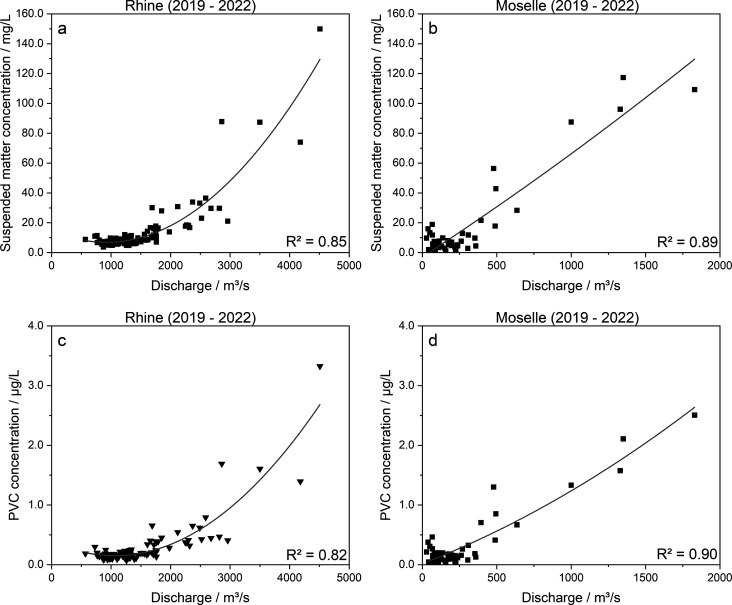
Relationship of SM concentration (mg/L; (a) Rhine, (b) Moselle) and PVC concentration (μg/L; (c) Rhine, (d) Moselle) to discharge (m^3^/s) on the respective sampling day. Gray lines represent quadratic fits.

In conclusion, these results confirm that SM and PVC show a similar scaling relationship. Discussions on sediment-discharge scaling often focus on sediment sources and transport pathways.^68–74^ In this context, our results indicate that SM and PVC share similar sources and transport pathways. The fact that SM mostly stems from erosive arable land and remobilization from the river channel may suggest that the majority of transported PVC MP is not caused by point sources, but rather by diffuse sources, similar to SM.^68,75^ In addition, CSOs may act as an event-driven and discharge-dependent source of PVC microplastics. Although not diffuse in the classical hydrological sense, CSOs are triggered by rainfall intensity thresholds and can exhibit nonlinear relationships with discharge, similar to SM. Their contribution may be particularly relevant in urbanized areas and during short-term high flow events.^20,76,77^

#### 3.1.2 Suspended PVC Loads

PVC MP loads (with a 95% confidence interval) for both rivers were calculated based on the biweekly and four-weekly samples for the Rhine (Figure 4) and the Moselle (Figure 5): For the Rhine annual PVC loads of 19 ± 2 t (2019), 10 ± 1 t (2020), 38 ± 4 t (2021), and 15 ± 2 t (2022) were determined. The annual loads of the Moselle were 2.0 ± 0.2 t (2019), 14 ± 2 t (2020), 17 ± 2 t (2021), and 2.8 ± 0.3 t (2022). Interannual variability mainly results from the variability of the mean annual discharge rates and SM concentrations, which are strongly related to the discharge. High PVC MP loads were particularly prevalent during high discharge events, which in turn also have a very strong influence on the annual loads of PVC. On average, 80% of the total PVC load in the Rhine were transported in only 35 ± 10% of the time. For the Moselle, the 80% share of load transport occurred in 20 ± 11% of the time. The observed exceedance times are in the same order of magnitude as the exceedance times of the SM transport, in accordance with the similar concentration–discharge relationship of SM and PVC. This illustrates the pivotal role of flood events for PVC MP loads in the rivers studied. The frequency of high discharge events with high loads of SM, therefore, largely determines the PVC load prevailing in a given year. High discharge events with high loads of SM occur more frequently in late winter and early spring in Central Europe,^78^ which is reflected in our observations on the seasonal behavior of PVC loads for both rivers: On both rivers, most flood events occur during the meteorological winter months from December to February and in March. This is due to the snowmelt and heavy rain events that are typical of this season for both rivers. Furthermore, occasional smaller flood events can be observed during spring (Rhine 2019, 2022; Moselle 2020, 2022) and the summer months (Rhine 2019–2021; Moselle 2021). Modeled seasonal variability in plastic concentrations and loads on a global level, as cited in the literature, has shown that the bulk of MP is transported during periods of heavy rainfall and that increased MP loads occur in European rivers in the period from November to May.^78^ In practical studies, a seasonally increased concentration of MP in watercourses was also observed during the wet season due to storm and rain events, such as monsoon periods.^39,79^ In addition, it was shown that the wet season and associated high discharge rates accounted for up to 81% of the total MP load (by weight).^39^ Both, the modeling and the observations gained in the practical studies are consistent with our observations of seasonal PVC concentrations and loads.

**Figure 4:**
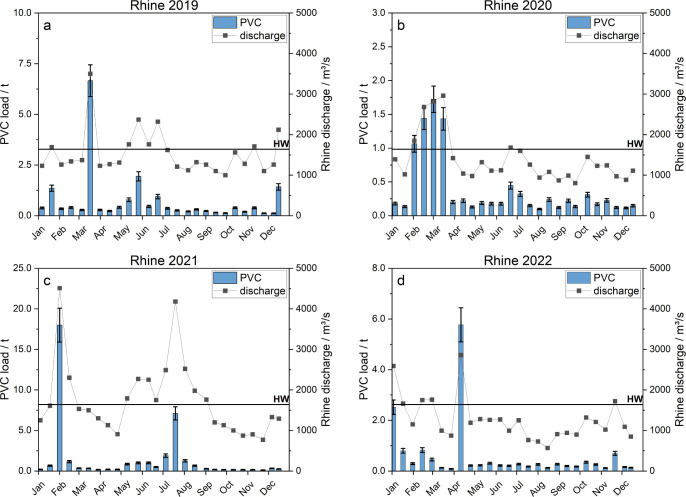
Calculated transported PVC (blue bars) for the Rhine River from 2019 to 2022 (a–d). Corresponding discharge rates are added as a dot/line diagram. Here, lines were inserted for visual purposes only and do not reflect the actual discharge trajectory. The solid black line represents the high water (HW) mark.

**Figure 5:**
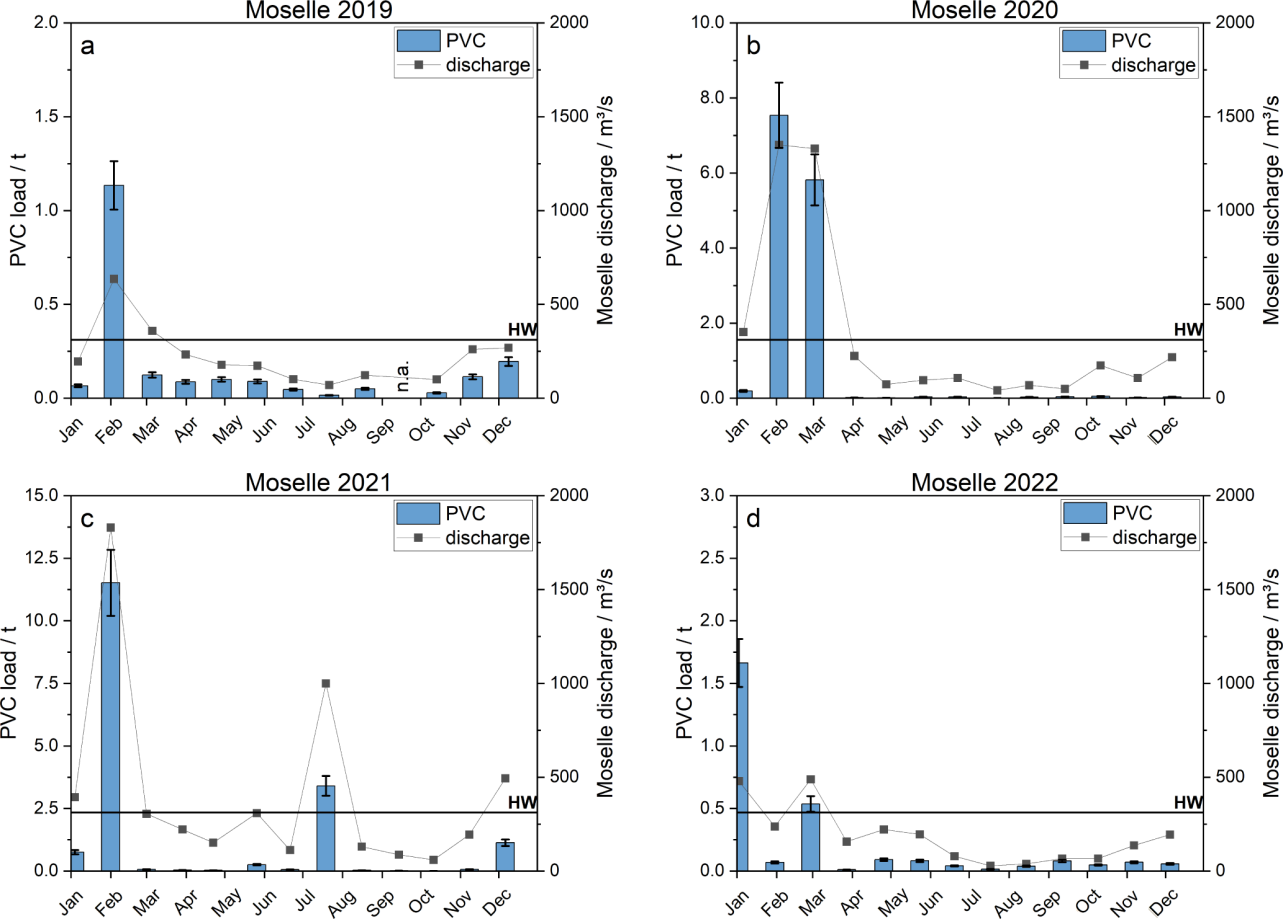
Calculated transported PVC (blue bars) for the Moselle River from 2019 to 2022 (a–d). Discharge rates measured on the respective sampling day are added as a dot/line diagram. Here, lines were inserted for visual purposes only and do not reflect the actual discharge trajectory. The solid black line represents the high water (HW) mark.

To date, the number of publications that calculate MP loads is limited and even lower for PVC.^17,33,38–42,80^ These studies determined MP loads using optical methods (such as FT-IR) for particle counting and mainly focused on polymer types other than PVC, which makes comparison with our mass-based PVC results difficult in several respects. Van der Wal et al. determined a PVC MP load of 0.5 t/a in the Rhine River in 2014 (total estimated MP load: 31 t/a).^41^ Despite the higher cut-off of 330 μm due to the use of filter nets by Van der Wal et al., our specific PVC load is in the same order of magnitude. It should also be mentioned that our sampling using CFC differs in the range of particles covered: While Van der Wal et al. discriminate particles <330 μm using filter nets, we cover a particle size range from 1 mm to 1 μm by using CFC sampling.^81^ Total MP loads from Eo et al.^39^ ranged between 53.3 t/a and 630 t/a for the Nakdong River in South Korea (determination via FT-IR; size range: 5 mm–20 μm). Here, PVC accounted for 1.1% to 5.4%, which in turn corresponds to the calculated load range in our study. However, it should be emphasized that this study investigated a different river with different discharge characteristics and a different sampling method. Direct comparability is therefore very limited.^52^ This underscores an urgent need for more systematic studies on microplastic concentrations and loads to expand our understanding of the microplastic fate in river systems and to explore measures which reduce their loads. Sampling limited to several days (such as the 10-day sampling period applied by Van der Wal et al.) is a too short period and insufficient for load estimations, as factors such as discharge fluctuations are not properly reflected. Monitoring needs to cover longer time spans and different discharge conditions to attain a more precise MP load calculation. In addition, high interannual variabilities require measurements over several years to develop robust monitoring methods for MP load estimation, as evidenced by the PVC load results in our study. Furthermore, our findings underscore the critical need for plastic pollution management policies including mitigation measures aimed at reducing potential sources of macro- and microplastics. Effective river management strategies could include enhanced rainwater overflow basin and (industrial) wastewater treatment standards to capture plastics before entering rivers and streams, regular monitoring programs to identify the diffuse emission hotspots, and public awareness campaigns to reduce plastic waste at its source. In addition, riparian-zone restoration or the integration of natural filtration systems such as wetlands could help to trap plastics before they reach major rivers such as the Moselle and Rhine. Collaborative governance, involving both local communities and business, is essential to implement these measures sustainably and equitably.

#### 3.1.3 Long-Term Spatial and Temporal PVC Trends

In addition to seasonal trends of PVC concentrations and loads, it is also interesting to consider longer periods of time. For this purpose, annual composite SM samples were collected on the Rhine every two years over a period of 17 years (2006–2022; exceptions: additional sampling in 2019 and 2021 for Koblenz; annual sampling from 2006 to 2015 for Iffezheim) in Weil, Iffezheim, Koblenz, and Bimmen and were analyzed for their PVC MP concentrations. Results shown in Section 3.1.1 indicate that PVC concentrations in SM for the Rhine at Koblenz constantly remain at the same level (Figures S.4 and 5). Building upon this fact, annual composite samples were now used to calculate loads. To this end, loads were determined from the respective triplicate PVC concentrations and annual SM loads (Table S.7).

The trend analysis revealed a significant nonlinear decreasing trend for Weil with an overall decrease of −48% (0.71 t/a; *p* = 0.03) (Figure 6 and Table S.7). For Iffezheim, Koblenz, and Bimmen significant linear trends (*p* = < 0.02) for PVC loads were observed. For Iffezheim an overall decrease of −58% (−1.38 t/a) was calculated, while Koblenz and Bimmen showed overall decreases of −38% (0.79 t/a) and −55% (1.72 t/a), respectively. The decrease in PVC loads over the years can be explained by the decreasing SM loads over the observed years in the Rhine despite a constant PVC concentration level (Table S.7) at the respective sites. Declining SM loads are attributed to decreased sediment supply, likely caused by reduced connectivity in the river network, and lower nutrient and organic matter levels.^70^ This is consistent with our observations indicating that PVC MP and SM show similar behaviors, as set out in the previous section.

**Figure 6:**
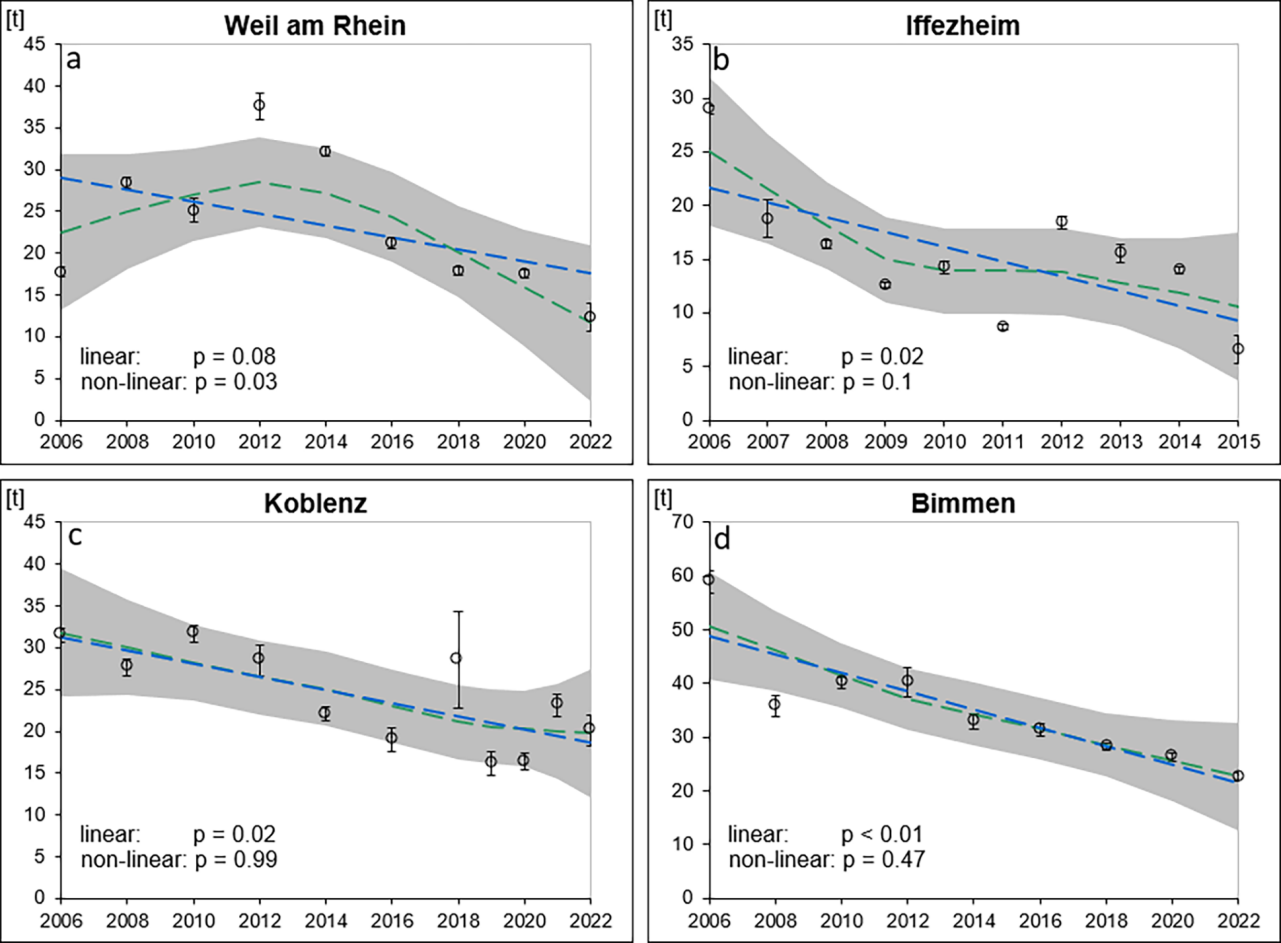
LOESS trend analysis of the PVC loads at Weil (a), Iffezheim (b), Koblenz (c), and Bimmen (d) from 2006 to 2022 (2006–2015 for Iffezheim). Blue dashed line: linear regression. Green dashed line: LOESS trend calculation. Gray area: error band of the 95% confidence interval.

Considering the trend of PVC concentrations over the studied period (Figure S.7), there were no significant increases/decreases at the relevant sites, and only slight fluctuations occurred. As PVC concentrations remained largely stable, temporal variations in PVC loads were mainly influenced by discharge similar to SM. Thus, SM load behavior may serve as an indicator of PVC load behavior, but not as a causative factor. In terms of spatial distributions, the mean PVC concentration (values are reported with 95% confidence intervals) at Weil was 29 ± 1 μg/g, while at Iffezheim (22 ± 0 μg/g) and Koblenz (17 ± 1 μg/g) lower concentrations were determined. Although both SM and PVC loads are higher in Koblenz compared to Iffezheim (Table S.7), the SM introduced between Iffezheim and Koblenz is less contaminated with PVC. This results in a dilution effect, reducing the overall concentration of PVC in the SM despite the increasing load. PVC concentrations at Bimmen were elevated (31 ± 1 μg/g), which means that more PVC was released into the Rhine during the period considered. Comparing mean PVC loads of all locations (Weil: 23 ± 6 t; Iffezheim: 15 ± 4 t; Koblenz: 24 ± 4 t; Bimmen: 35 ± 8 t; values are reported with 95% confidence intervals) reveals an increase in the load transported along the Rhine, starting at Iffezheim. Although general SM loads (Table S.7) increased on their way downstream the Rhine and were thus higher at each subsequent sampling site in all years. In general, the specific PVC load in Weil is higher than the load in Iffezheim in all comparable years. The SM loads (Table S.7) are also higher in these years, which indicates that sinks for SM and PVC have a significant impact on the loads between Weil and Iffezheim. Furthermore, results showed that PVC MP concentrations and loads depend on the sampled location and are influenced by diverse sources, including industrial sites and wastewater discharges, and sinks such as floodplains located between successive monitoring stations, as Mani et al. reported in 2016.^82^

### 3.2 Limitations, Implications, and Future Research Directions

This study successfully monitored PVC MP in riverine environments, marking a significant step in understanding their presence and behavior in aquatic systems. PVC was chosen as the focus of this research because it is the third most-produced plastic globally and contains the highest proportion of ecotoxicologically relevant additives among common polymers. This study represents the first long-term systematic study of PVC MP in freshwater systems, providing valuable insights into its transport and distribution. Nevertheless, it is crucial to consider the methodological limitations associated with the CFC sampling of SM and MP. In our study, the sampling was conducted at a water depth of approximately 0.5 m below the water surface, in close proximity to the river banks (5 m distance). However, this limited spatial coverage does not reflect the actual particle distribution in the river cross section, which can vary considerably.^43^ Furthermore, the cross-sectional position in the watercourse can influence the results, since the transport of SM in areas close to the banks differs from SM transport in the middle of a river, where higher flow velocities and turbulences prevail. An additional consideration relates to the density-specific behavior of different polymers in the water column. PVC, with a density of approximately 1.4 g/cm^3^, may tend to accumulate in deeper layers of the river, particularly under calm flow conditions or low turbulences.^28,60^ Although the dynamic flow conditions in rivers can keep such particles in suspension throughout the water column, we cannot totally exclude that the near-surface sampling underrepresents deeper PVC concentrations. However, we are confident that this does not influence the comparability of our results, since the sampling approach was exactly the same from 2006 to 2022. In contrast, polymers such as PE and PP, which are less dense than water, may preferentially accumulate closer to the surface or exhibit different transport dynamics. This aspect should be considered in future studies, particularly when designing vertically resolved sampling strategies.

A further methodological limitation is that the sampling was nonisokinetic as the flow rate of the sampling pump was set to approximately 15 L/min resulting in an inflow velocity of approximately 0.007 m/s, which is far smaller than the flow velocity of around 1 m/s observed in the Rhine and Moselle.^88^ Given the inertia of the particles, SM in the sample tends to be overestimated when pumping at a velocity below the velocity of the river flow. Conversely, greater accuracy in the estimation of SM would be achieved when pumping velocity equals flow velocity.^62^ Therefore, isokinetic sampling could ensure greater accuracy in that it would better reflect SM and MP concentrations of the watercourse. Unfortunately, the implementation of isokinetic sampling in rivers such as the Rhine is challenging due to the variable flow velocity and turbulence, as well as the complex technical requirements.

Furthermore, sampling was conducted at fixed regular intervals, either biweekly or four-weekly. This restricted temporal resolution resulted in inadequate documentation of fluctuations in the SM and MP loads, particularly during high flow events. Consequently, calculated annual PVC loads are based on averaged discharge rates, which might underestimate the true MP load. Future research is recommended to focus on optimizing the sampling interval to identify an appropriate balance between the feasibility of sampling and analysis and the accuracy of the load estimates. This is of particular importance with regard to spontaneous extreme events such as floods, which can cause significant fluctuations in the loads of SM and MP.

Eventually, it would be beneficial for future studies to consider a wider range of plastics including PE, PP and PS, to gain a more comprehensive understanding of MP loads in watercourses. However, it should be noted that these polymers differ structurally from PVC and do not generate any specific marker compounds in the C-IC analysis. Therefore, their quantitative analysis requires alternative analytical approaches such as pyrolysis GC-MS, which allows for polymerspecific identification and quantification based on characteristic pyrolysis fragments.^43^

In terms of harmonization, the use of a PLE coupled with C-IC offers several advantages. This method is not only robust and sensitive enough for environmental PVC MP analysis but also features simple handling and cost-effective instrumentation. As such, it is well-suited for routine applications, even in smaller laboratories and research institutes, making it a promising tool for standardized monitoring efforts. World-wide harmonized protocols for sampling and analyzing plastic loads would help to enhance the comparability of results gained in different studies.

## Supplementary Material



## Notes

Sample preparation and the extraction method, experimental setup, analytical method, PVC concentrations for all measured samples and sampling parameters including sampled volume, sample mass, discharge and turbidity; additional PVC concentration vs time graphics for all monitored years and LOESS trend analysis graphic for PVC concentrations of the long-term sampling (PDF)
