# Supplementary material for: Occurrence, Temporal Variability, and Loads of Poly(vinyl chloride) in the Rhine and Moselle
Source: Environ Sci Technol. 2025 Jul 7;59(28):14649–59. doi: 10.1021/acs.est.5c03236 (PMC12288088; doi:10.1021/acs.est.5c03236)
Supplement: Supplementary file 1 [file es5c03236_si_001.pdf]

Supporting Information

Occurrence, temporal variability and loads of poly(vinyl chloride) in the Rhine and Moselle

Jan Kamp[1,2], Georg Dierkes[1], David Range[1], Thomas Hoffmann[1,2], Thomas A. Ternes[1,2]*

[1] Federal Institute of Hydrology, Am Mainzer Tor 1, 56068, Koblenz, Germany

[2] University of Koblenz, Universitätsstraße 1, 56070, Koblenz, Germany

* To whom correspondence should be addressed: ternes@bafg.de

Summary: 16 pages, 2 sections, 7 figures, 7 tables.

Contents

S1. Sample preparation and extraction S3
S2. Quantification *via* Combustion ion chromatography S3
Table S.1: Experimental parameters and instrumental parts of the C-IC system S5
Table S.2: Sampling details, PVC concentrations (µg/g & µg/L) and calculated PVC load of the Rhine and Moselle Monitoring 2019. ....S6
Table S.3: Sampling details, PVC concentrations (µg/g & µg/L) and calculated PVC load of the Rhine and Moselle Monitoring 2020. ....S7
Table S.4: Sampling details, PVC concentrations (µg/g & µg/L) and calculated PVC load of the Rhine and Moselle Monitoring 2021. ....S8
Table S.5: Sampling details, PVC concentrations (µg/g & µg/L) and calculated PVC load of the Rhine and Moselle Monitoring 2022. ....S9

Table S.6: Spearman rank correlation coefficients and p-values for the river Rhine and Moselle. S10
Table S.7: Trend analysis PVC concentrations (µg/g) and loads (t) of all annual composite samples (2006 – 2022) for Weil, Iffezheim (only 2006-2015), Koblenz and Bimmen...........S10

Figure S.1: Calibration curve for PVC combustion after extraction. Calibration range was 0.01 mg/g to 5.0 mg/g...S3
Figure S.2: PVC concentrations (µg/L) for the years 2019 to 2022 (a - d) compared to the suspended matter concentrations (mg/L) from the Rhine. S11
Figure S.3: PVC concentrations (µg/L) for the years 2019 to 2022 (a - d) compared to the suspended matter concentrations (mg/L) from the Moselle. S12
Figure S.4: Comparison of the behavior of the PVC concentration (mg/g) over the years 2019 to 2022 (a – d) in the collected suspended matter samples and the suspended matter concentration (mg/L) in the Rhine. S13
Figure S.5: Comparison of the behavior of the PVC concentration (mg/g) in the Moselle over the years 2019 to 2022 (a – d) in the collected suspended matter samples and the suspended matter concentration (mg/L). October 2019 was not applicable (n.a.). S14
Figure S.6: Logarithmic scale depiction of the SM concentration (mg/L; a) Rhine, b) Moselle) and the PVC concentration (µg/L; c) Rhine, d) Moselle) against the discharge (m³/s) of the respective sampling day. S15
Figure S.7: LOESS trend analysis of the PVC concentrations (µg/g) at Weil (a), Iffezheim (b), Koblenz (c) and Bimmen (d) from 2006 to 2022. Blue dashed line: linear regression. Green dashed line: LOESS trend calculation. Grey area: error band of the 95% confidence interval. S16

References S16

S1. Sample preparation and extraction

Sediment samples were fractionated by wet sieving (5-1 mm, 1 mm - 500 µm, 500-100 µm, 100-50 µm, 50-10 µm). All sediments, SM samples and sample fractions were freeze-dried and ground for homogenization using a planet mill (Fritsch, Idar-Oberstein, Germany). For PLE (Pressurized Liquid Extraction) a SpeedExtractor E-916 system (BÜCHI Labortechnik GmbH, Essen, Germany) was used. Extractions were carried out according to *Dierkes et al.*[1]: All samples were washed in a clean-up step under pressure (100 bar) with methanol at 100 °C (discarded) and then extracted using tetrahydrofuran (THF) at 185 °C and 100 bar (collected). 200 mg silica gel was placed in the collection vessels, which served as a sorption medium for the PVC after evaporation of the THF. 1.0 g of each dried sediment and SM sample was used for extraction. For calibration, additive-free PVC (PyroPowders.de, Erfurt, Germany) with concentrations in sea sand of 5.0, 2.5, 1.0, 0.5, 0.1, 0.05 and 0.01 mg/g was extracted and analyzed in triplicate. Figure S1 shows the calibration curve with standard deviations for all calibration points. Based on mean extraction blank PVC concentration with the two-sided confidence interval (CI) 95% (n = 45; $t_{(p = 0.95;\ n-1 = 44)} = 2.017$) was calculated. The limit of quantification (LOQ) was defined as the upper CI limit plus 2·σ. A LOQ of 7.3 µg/g for the PVC method via PLE combined with C-IC. A concentration of 3.1 ± 1.6 µg/g PVC was determined as background contamination.

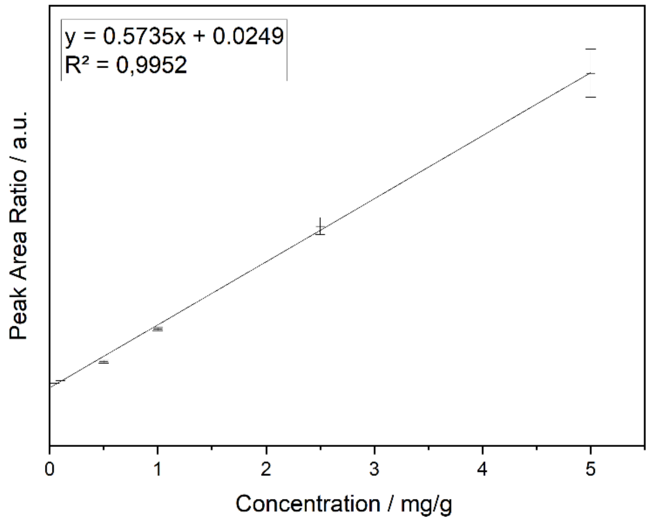

Figure S. 1: Calibration curve for PVC combustion after extraction. Calibration range was 0.01 mg/g to 5.0 mg/g.

S2. Quantification *via* Combustion ion chromatography

All C-IC (combustion ion chromatography) measurements were done according to *Kamp et al.*[2]. Exact 20 mg of each silica gel extract were combusted at 1000 °C for 18 min *via* an ASC-240S autosampler and an AQF-2100H combustion oven (Mitsubishi Chemical Analytech, Yamatoshi, Japan). A GA-210 absorption unit (Mitsubishi Chemical Analytech) was used for absorption. An 881 Compact IC pro, equipped with a conductivity detector from Metrohm (Filderstadt, Germany), was used for the detection of chloride. For absorption, 10 mL MilliQ water was used as absorbent solution with 5.0 mg/L phosphoric acid as internal standard. For IC, MilliQ water mixed with 3.2 mmol/L Na_2CO_3 & 1.0 mmol/L $NaHCO_3$ as eluent and 0.05 mmol/L phosphoric acid as suppressor solution were used. The PVC content was determined *via* the chloride concentration contained in the absorbent solution. Further detailed setup parameters are listed in Table S.1.

Table S. 1: Experimental parameters and instrumental parts of the C-IC system.

Instrumental parameters	Value
**Combustion**	
Instrument	ASC-240S (auto sampler), AQF-2100H (combustion)
Furnace temperature	900 - 1000 °C
Combustion time	18 min
Carrier gas	200 mL/min Ar (5.0, Linde GmbH, Pullach, Germany)
	400 mL/min O_2 (5.0, Linde GmbH)
**Absorption**	
Instrument	GA-210
Absorption solution	300 mg/L H_2O_2, 5 mg/L phosphate, MilliQ water
Absorption solution volume	10 mL
**Ion chromatography**	
Instrument	881 Compact IC pro
Column	Metrosep A Supp 5 -150/4.0
Mobile phase	3.2 mmol/L Na_2CO_3; 1.0 mmol/L $NaHCO_3$; isocratic
Suppressor	250 mmol/L H_3PO_4
Flow rate	0.7 mL/min
Oven temperature	45 °C
Injection volume	100.0 µL
Detector	Conductivity detector

Table S. 2: Sampling details, PVC concentrations (µg/g & µg/L) and calculated PVC loads with standard errors of the Rhine and Moselle Monitoring 2019.

Sampling location	Date	PVC conc. / µg/g	Suspended matter / mg/L	Sampled volume / L	Sample mass / g	PVC-conc. / µg/L	Discharge / m³/s	Transport / mg/s	PVC load / t	Turbidity / TE/F
Moselle	07.01.2019	27 ± 3	4.68	7009	25.30	0.0983	196	24.77	2 ± 0	5
	04.02.2019	26 ± 3	28.35	6078	157.77	0.6672	636	468.80		n.a.
	06.03.2019	29 ± 3	4.44	5834	25.89	0.1271	359	46.22		n.a.
	01.04.2019	28 ± 3	5.19	6879	31.98	0.1299	233	33.86		7
	29.04.2019	29 ± 3	7.26	6776	42.16	0.1781	178	37.48		7
	27.05.2019	24 ± 3	8.05	6762	46.49	0.1681	173	33.42		6
	24.06.2019	24 ± 3	7.41	7059	48.43	0.1626	101	17.96		5
	22.07.2019	24 ± 3	3.46	7464	23.92	0.0782	70	5.81		5
	19.08.2019	36 ± 4	4.32	8578	32.36	0.1343	123	19.13		9
	14.10.2019	25 ± 3	4.31	7175	28.27	0.0980	100	10.78		5
	11.11.2019	22 ± 3	7.66	7696	55.48	0.1574	261	43.98		10
	09.12.2019	21 ± 2	12.92	7359	90.19	0.2616	269	72.99		14
Rhine	07.01.2019	23 ± 3	11.19	6877	70.79	0.2317	1230	316.57	19 ± 2	8
	21.01.2019	22 ± 2	30.12	4504	135.66	0.6534	1690	1119.86		28
	04.02.2019	23 ± 3	9.78	6731	60.94	0.2079	1260	283.42		7
	18.02.2019	22 ± 3	11.36	5341	60.66	0.2533	1340	334.89		9
	06.03.2019	23 ± 3	7.48	6414	44.33	0.1591	1370	235.69		7
	18.03.2019	18 ± 2	87.39	3347	292.50	1.6064	3500	5505.57		83
	01.04.2019	16 ± 2	12.03	7158	80.91	0.1811	1230	236.75		7
	15.04.2019	21 ± 2	7.29	6878	50.14	0.1512	1270	194.42		5
	29.04.2019	25 ± 3	10.43	6887	66.37	0.2443	1310	341.58		8
	13.05.2019	23 ± 3	16.02	6589	105.53	0.3744	1760	648.49		13
	27.05.2019	20 ± 2	33.88	4188	133.38	0.6500	2370	1605.91		30
	11.06.2019	21 ± 2	10.30	6827	70.30	0.2192	1760	380.69		8
	24.06.2019	20 ± 2	16.77	5379	84.46	0.3182	2320	778.13		13
	08.07.2019	21 ± 2	8.85	6908	61.11	0.1868	1620	301.08		5
	22.07.2019	23 ± 3	7.83	6570	46.48	0.1626	1210	217.91		5
	07.08.2019	23 ± 3	6.73	7788	52.41	0.1547	1120	173.36		5
	19.08.2019	24 ± 3	8.06	6917	51.54	0.1764	1320	255.34		5
	02.09.2019	20 ± 2	7.64	6291	48.05	0.1550	1260	192.53		6
	16.09.2019	20 ± 2	5.94	6077	32.74	0.1072	1100	130.68		4
	30.09.2019	21 ± 2	5.15	7570	39.01	0.1100	999	108.04		30
	14.10.2019	17 ± 2	12.18	5956	66.93	0.1865	1560	323.01		10
	28.10.2019	16 ± 2	7.97	6869	54.77	0.1277	1280	163.23		7
	11.11.2019	16 ± 2	11.72	6870	74.45	0.1720	1710	320.66		10
	25.11.2019	16 ± 2	5.89	7046	41.48	0.0955	1100	103.66		5
	09.12.2019	16 ± 2	4.92	6512	29.27	0.0716	1260	99.19		5
	16.12.2019	18 ± 2	30.81	5992	184.61	0.5435	2120	1175.71		23-35

Table S. 3: Sampling details, PVC concentrations (µg/g & µg/L) and calculated PVC loads with standard errors of the Rhine and Moselle Monitoring 2020.

**Sampling location**	**Date**	**PVC conc. / µg/g**	**Susp. matter / mg/L**	**Sampled volume / L**	**Sample mass / g**	**PVC-conc. / µg/L**	**Discharge / m^3/s**	**Transport / mg/s**	**PVC load / t**	**Turbidity / TE/F**
Moselle	06.01.2020	21 ± 2	9.76	7529	67.57	0.1844	353	72.35	14 ± 2	11
	03.02.2020	19 ± 2	117.32	2592	287.64	2.1071	1350	3009.26		106
	02.03.2020	17 ± 2	96.07	2592	237.21	1.5752	1330	2172.14		64
	02.04.2020	18 ± 2	1.61	16936	24.69	0.0265	225	6.52		n.a.
	27.04.2020	25 ± 3	2.65	8248	19.73	0.0606	74	4.90		3
	26.05.2020	21 ± 2	6.91	8139	53.09	0.1349	96	13.93		4
	22.06.2020	18 ± 2	7.27	5434	37.03	0.1247	108	14.13		5
	23.07.2020	20 ± 2	2.04	17062	38.20	0.0456	42	1.71		2
	17.08.2020	29 ± 3	5.77	3212	16.72	0.1510	69	11.55		16
	14.09.2020	25 ± 3	13.27	5689	69.43	0.3064	50	16.59		n.a.
	12.10.2020	20 ± 2	5.39	6848	32.05	0.0923	175	18.87		5
	10.11.2020	17 ± 2	3.38	6191	19.24	0.0514	108	6.21		3
	07.12.2020	16 ± 2	3.82	20019	71.98	0.0582	219	13.39		3
Rhine	06.01.2020	18 ± 2	5.95	6895	36.94	0.0940	1390	148.87	10 ± 1	6
	20.01.2020	19 ± 2	5.78	6979	40.33	0.1112	1020	112.02		5
	03.02.2020	17 ± 2	27.98	4275	112.52	0.4526	1850	879.97		35
	17.02.2020	15 ± 2	29.64	4585	135.91	0.4452	2680	1191.53		34
	02.03.2020	17 ± 2	29.72	4771	132.81	0.4683	2820	1424.78		29
	16.03.2020	19 ± 2	21.07	6134	129.27	0.4065	2960	1184.98		19
	30.03.2020	19 ± 2	6.22	6889	39.50	0.1081	1420	167.82		n.a.
	14.04.2020	21 ± 2	8.39	6928	58.12	0.1795	1040	183.24		5
	27.04.2020	18 ± 2	6.04	6189	34.33	0.0991	977	106.22		4
	11.05.2020	19 ± 2	6.19	6238	38.59	0.1177	1320	155.25		5
	25.05.2020	19 ± 2	6.96	6426	40.12	0.1190	1110	146.79		4
	08.06.2020	18 ± 2	7.29	6670	48.60	0.1284	1120	146.97		4
	22.06.2020	22 ± 3	9.97	6831	63.76	0.2072	1680	368.49		6
	06.07.2020	20 ± 2	8.32	7374	61.35	0.1670	1600	266.24		5
	20.07.2020	18 ± 2	5.46	7207	35.87	0.0879	1260	123.83		4
	03.08.2020	17 ± 2	5.11	6586	33.63	0.0863	939	81.57		4
	17.08.2020	19 ± 2	9.62	7611	67.78	0.1669	1080	197.40		6
	31.08.2020	19 ± 2	6.08	7014	42.66	0.1172	870	100.50		4
	14.09.2020	19 ± 2	9.73	6548	57.97	0.1660	988	182.65		7
	24.09.2020	17 ± 2	8.26	6829	56.41	0.1408	803	112.76		n.a.
	12.10.2020	18 ± 2	9.84	7004	63.67	0.1608	1450	256.82		8
	26.10.2020	19 ± 2	6.02	6482	38.99	0.1118	1230	140.69		4
	09.11.2020	18 ± 2	8.36	6344	48.40	0.1392	1240	186.60		5
	23.11.2020	18 ± 2	5.81	6393	37.16	0.1058	972	101.65		4
	07.12.2020	19 ± 2	5.70	6111	31.90	0.0974	886	95.95		n.a.
	17.12.2020	19 ± 2	5.80	7994	46.36	0.1129	1110	122.32		4

Table S. 4: Sampling details, PVC concentrations (µg/g & µg/L) and calculated PVC loads with standard errors of the Rhine and Moselle Monitoring 2021.

Sampling location	Date	PVC conc. / µg/g	Susp. matter / mg/L	Sampled volume / L	Sample mass / g	PVC-conc. / µg/L	Discharge / m³/s	Transport / mg/s	PVC load / t	Turbidity / TE/F
Moselle	04.01.2021	33 ± 4	21.61	3796	82.01	0.7047	394	280.97	17 ± 2	22
	01.02.2021	23 ± 3	109.23	3817	416.92	2.5053	1830	4597.49		137
	01.03.2021	28 ± 3	2.83	18983	53.80	0.0782	305	24.17		3
	29.03.2021	27 ± 3	2.43	17366	42.12	0.0652	222	14.57		4
	22.04.2021	25 ± 3	2.87	16583	47.53	0.0712	151	10.83		3
	26.05.2021	27 ± 3	11.84	8270	97.91	0.3243	309	98.78		9
	21.06.2021	26 ± 3	7.74	19149	148.20	0.1989	112	22.54		11
	19.07.2021	15 ± 2	87.54	433	37.90	1.3323	1000	1313.10		94
	16.08.2021	22 ± 3	3.93	18350	72.13	0.0870	130	11.24		6
	14.09.2021	28 ± 3	1.69	15892	26.85	0.0475	87	4.12		1
	11.10.2021	26 ± 3	1.44	17644	25.42	0.0373	59.4	2.22		3
	08.11.2021	22 ± 3	6.03	7095	42.80	0.1318	194	25.74		8
	06.12.2021	20 ± 2	42.79	6902	295.33	0.8521	494	422.77		30
Rhine	04.01.2021	22 ± 3	6.30	6457	40.69	0.1414	1250	173.25	38 ± 4	5
	18.01.2021	24 ± 3	14.35	5812	83.41	0.3404	1610	554.48		15
	01.02.2021	22 ± 3	149.89	2032	304.58	3.3239	4510	14872.09		145
	15.02.2021	23 ± 3	18.19	5759	104.77	0.4111	2300	962.25		17
	01.03.2021	23 ± 3	8.47	6163	52.20	0.1983	1530	298.06		6
	15.03.2021	26 ± 3	7.28	5836	42.47	0.1922	1500	283.92		7
	29.03.2021	24 ± 3	4.79	6463	30.95	0.1165	1300	149.45		4
	12.04.2021	25 ± 3	6.38	7104	45.35	0.1612	1130	180.24		4
	26.04.2021	29 ± 3	6.51	6188	40.27	0.1856	907	171.23		4
	10.05.2021	24 ± 3	16.47	5896	97.09	0.3893	1790	707.55		11
	26.05.2021	20 ± 2	18.46	6206	114.57	0.3746	2270	838.08		10
	10.06.2021	21 ± 2	17.70	6376	112.86	0.3729	2250	836.33		6
	21.06.2021	19 ± 2	12.57	6702	84.25	0.2409	1750	417.95		5
	05.07.2021	19 ± 2	33.17	4334	143.76	0.6173	2490	1569.27		29
	19.07.2021	19 ± 2	74.00	2610	193.13	1.3935	4180	5877.08		75
	02.08.2021	18 ± 2	23.08	4299	99.21	0.4257	2520	1046.91		15
	16.08.2021	20 ± 2	13.89	6030	83.74	0.2806	1980	550.04		13-9
	02.09.2021	19 ± 2	7.10	6837	48.55	0.1339	1760	237.42		5
	14.09.2021	20 ± 2	7.00	7533	52.71	0.1431	1200	168.00		4
	27.09.2021	21 ± 2	6.09	6471	39.39	0.1294	1130	144.52		4
	11.10.2021	20 ± 2	6.97	6352	44.30	0.1386	1000	139.40		4
	25.10.2021	23 ± 3	7.36	6602	48.62	0.1710	872	147.61		4
	08.11.2021	25 ± 3	6.53	6590	43.00	0.1624	903	147.41		5
	23.11.2021	22 ± 3	6.73	6473	43.56	0.1511	769	113.86		4
	06.12.2021	21 ± 2	10.19	6060	61.77	0.2153	1330	284.61		8
	16.12.2021	20 ± 2	8.46	6243	52.82	0.1654	1290	218.27		7

Table S. 5: Sampling details, PVC concentrations (µg/g & µg/L) and calculated PVC loads with standard errors of the Rhine and Moselle Monitoring 2022.

Sampling location	Date	PVC conc. / µg/g	Susp. matter / mg/L	Sampled volume / L	Sample mass / g	PVC-conc. / µg/L	Discharge / m³/s	Transport / mg/s	PVC load / t	Turbidity / TE/F
Moselle	03.01.2022	23 ± 3	56.37	2013	113.47	1.3016	479	621.03	3 ± 0	66
	31.01.2022	26 ± 3	4.49	7820	35.09	0.1157	237	27.67		6
	28.02.2022	23 ± 3	17.80	4088	72.78	0.4118	489	200.20		25
	28.03.2022	25 ± 3	1.08	17304	18.64	0.0271	157	4.24		2
	26.04.2022	36 ± 4	4.26	14562	62.07	0.1514	221	33.89		n.a.
	24.05.2022	23 ± 3	7.03	14804	104.12	0.1639	196	31.69		n.a.
	20.06.2022	26 ± 3	7.52	7210	54.21	0.1969	79	15.45		5
	18.07.2022	22 ± 3	9.71	6445	62.61	0.2127	28	5.98		8
	15.08.2022	24 ± 3	15.98	4880	78.00	0.3759	38	14.57		26
	12.09.2022	25 ± 3	18.86	3796	71.58	0.4645	66	31.12		38
	10.10.2022	23 ± 3	11.76	7173	84.38	0.2677	67	18.12		11
	07.11.2022	20 ± 2	9.89	6538	64.67	0.1976	137	27.10		8
	05.12.2022	22 ± 2	5.05	7351	37.10	0.1090	194	21.55		5
Rhine	03.01.2022	22 ± 2	36.51	4404	160.79	0.7911	2590	2080.34	15 ± 2	36
	17.01.2022	24 ± 3	16.69	4448	74.23	0.4022	1660	664.93		14
	31.01.2022	23 ± 3	9.16	6400	58.62	0.2148	1150	242.28		6
	14.02.2022	22 ± 3	17.78	5013	89.12	0.3911	1750	684.53		21-28
	28.02.2022	20 ± 2	10.72	5396	57.82	0.2182	1760	377.34		11
	14.03.2022	22 ± 3	4.91	6075	29.81	0.1081	995	107.48		4
	28.03.2022	22 ± 3	3.78	7848	29.69	0.0828	872	72.52		2
	11.04.2022	19 ± 2	87.81	3679	323.04	1.6892	2860	4771.60		84
	25.04.2022	22 ± 2	6.95	6479	45.00	0.1500	1190	181.95		6
	09.05.2022	20 ± 2	7.37	7169	52.81	0.1474	1280	188.67		5
	23.05.2022	22 ± 3	9.23	7049	65.04	0.2043	1260	255.86		5
	07.06.2022	22 ± 3	6.52	6891	44.96	0.1440	1270	182.17		4
	20.06.2022	20 ± 2	8.75	6253	54.72	0.1752	995	174.13		5
	04.07.2022	18 ± 2	10.14	6445	65.38	0.1825	1250	228.15		84
	18.07.2022	17 ± 2	11.38	6436	73.23	0.1990	764	147.80		8
	01.08.2022	27 ± 3	11.02	6573	72.42	0.2976	727	216.31		8
	15.08.2022	21 ± 2	8.84	7075	62.51	0.1866	568	105.44		7
	29.08.2022	35 ± 4	7.03	6964	48.96	0.2472	909	223.66		4
	12.09.2022	23 ± 3	7.63	6657	50.80	0.1732	940	164.96		6
	26.09.2022	23 ± 3	7.16	7076	50.64	0.1613	898	147.88		6
	10.10.2022	20 ± 2	10.96	6599	72.33	0.2173	1320	289.34		8
	24.10.2022	21 ± 2	8.48	6725	57.06	0.1778	1210	215.48		7
	07.11.2022	20 ± 2	4.90	7636	37.40	0.0995	1020	99.96		4
	21.11.2022	21 ± 2	16.01	5509	88.19	0.3328	1720	578.28		14
	05.12.2022	22 ± 3	5.58	7003	39.06	0.1223	1090	133.81		4
	14.12.2022	23 ± 3	5.65	7070	39.92	0.1293	847	110.07		4

Table S. 6: Spearman rank correlation coefficients and p-values for the river Rhine and Moselle.

River	Correlation coefficient	p-value
Rhine	0.941	6.03×10^{-50}
Moselle	0.975	1.02×10^{-33}

Table S. 7: Trend analysis PVC concentrations (µg/g) and loads (t) with standard deviations of all annual composite samples (2006 – 2022) for Weil, Iffezheim (only 2006-2015), Koblenz and Bimmen.

Sampling location	Year	PVC conc. / µg/g	SM load / t	PVC load / t
Weil	2006	28 ± 1	642756	18 ± 1
	2008	27 ± 1	1067558	28 ± 1
	2010	29 ± 2	875963	25 ± 1
	2012	29 ± 1	1306276	38 ± 2
	2014	29 ± 1	1119566	32 ± 1
	2016	30 ± 1	697673	21 ± 1
	2018	28 ± 1	632616	18 ± 0
	2020	28 ± 1	613343	17 ± 0
	2022	32 ± 4	383259	12 ± 2
Iffezheim	2006	21 ± 1	815003	17 ± 0
	2007	23 ± 2	900856	21 ± 2
	2008	23 ± 1	689834	16 ± 0
	2009	23 ± 0	520768	12 ± 0
	2010	22 ± 1	596120	13 ± 1
	2011	22 ± 1	363121	8 ± 0
	2012	22 ± 1	763726	17 ± 1
	2013	22 ± 1	784511	17 ± 1
	2014	22 ± 0	693034	15 ± 0
	2015	23 ± 2	734321	17 ± 1
Koblenz	2006	15 ± 0	2158760	32 ± 1
	2008	20 ± 1	1388330	28 ± 1
	2010	19 ± 1	1681678	32 ± 1
	2012	18 ± 1	1596382	28 ± 2
	2014	17 ± 1	1305365	22 ± 1
	2016	14 ± 1	1368587	19 ± 1
	2018	19 ± 4	1530033	29 ± 6
	2019	16 ± 1	1037034	16 ± 1
	2020	16 ± 1	1018111	16 ± 1
	2021	14 ± 1	1626386	23 ± 1
	2022	18 ± 2	1113123	20 ± 2
Bimmen	2006	30 ± 1	1967390	59 ± 2
	2008	30 ± 2	1190210	36 ± 2
	2010	29 ± 1	1377848	40 ± 1
	2012	31 ± 2	1310650	40 ± 3
	2014	35 ± 2	945677	33 ± 1
	2016	30 ± 1	1040810	31 ± 1
	2018	31 ± 1	914942	28 ± 1
	2020	35 ± 1	753393	26 ± 1
	2022	32 ± 1	715142	23 ± 1

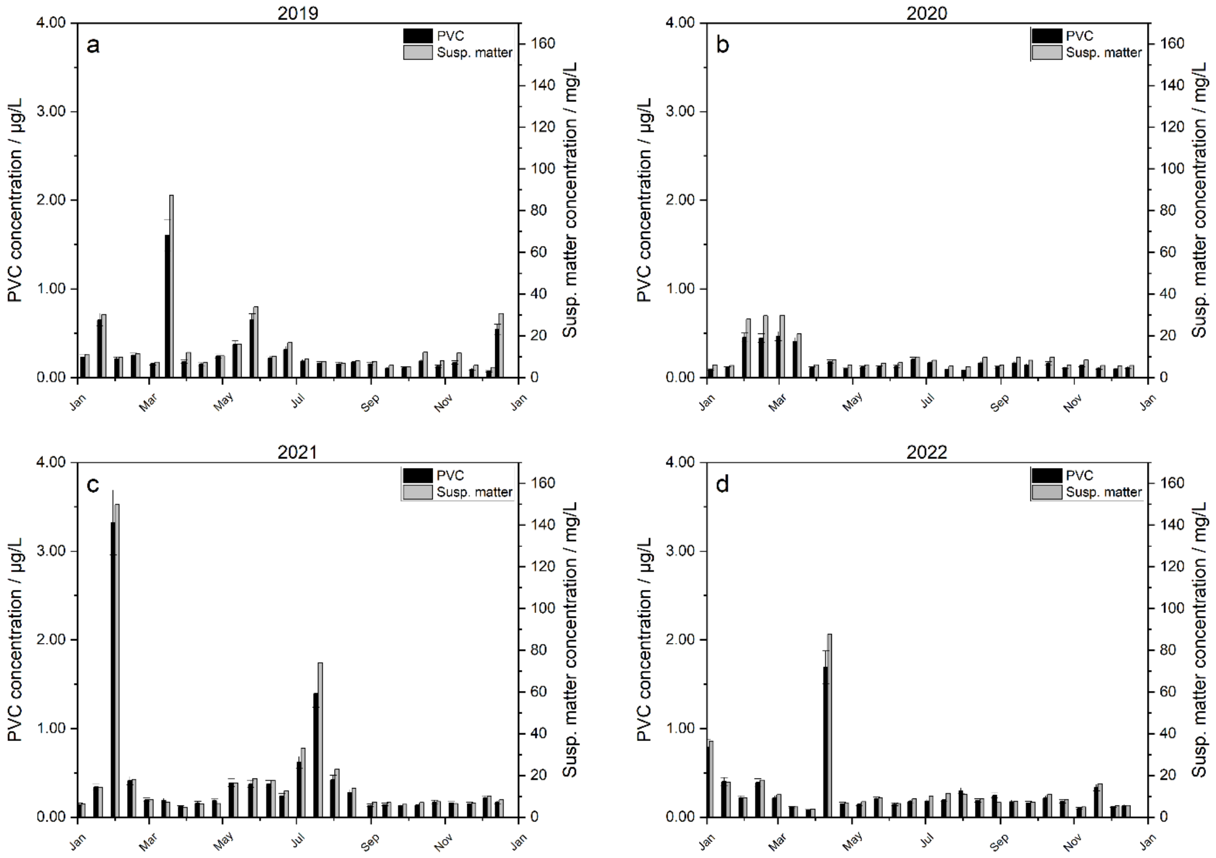

Figure S.2: PVC concentrations (µg/L) for the years 2019 to 2022 (a - d) compared to the suspended matter concentrations (mg/L) from the Rhine.

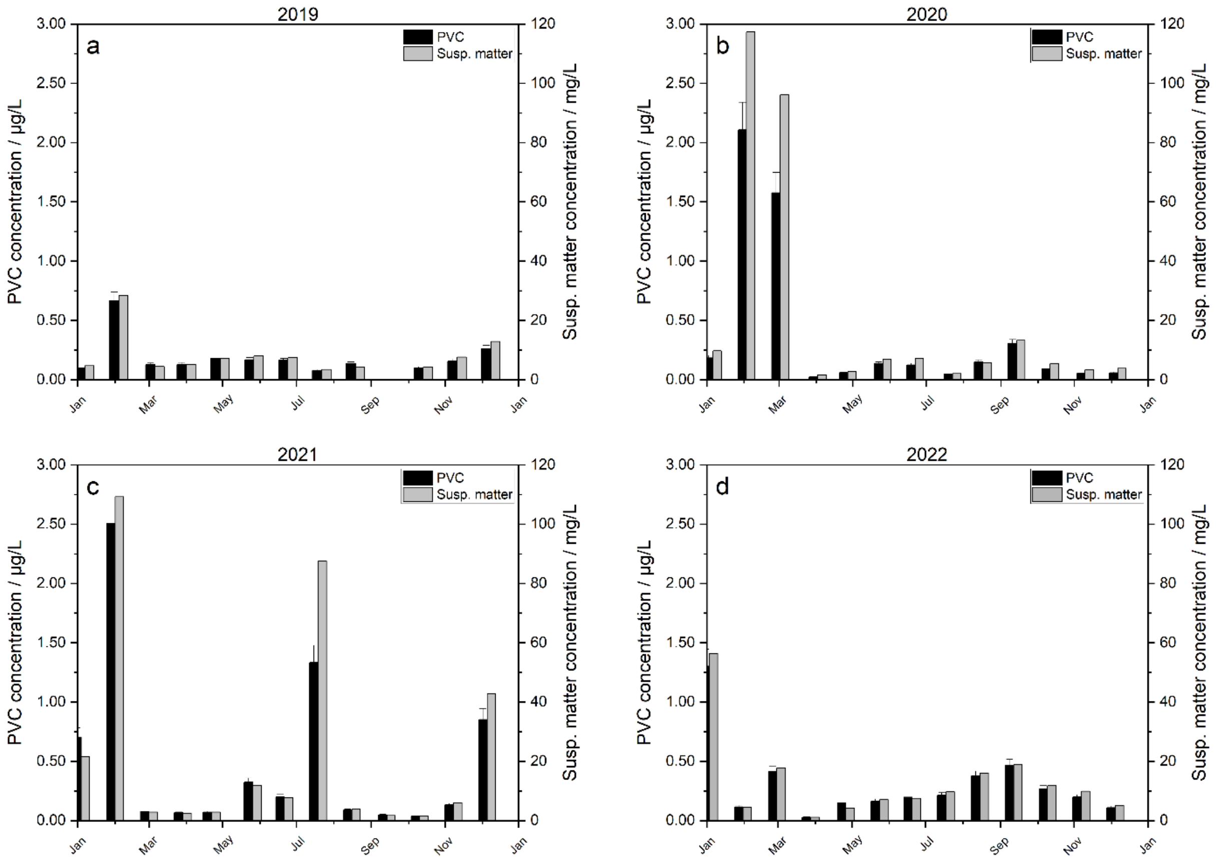

Figure S.3: PVC concentrations (µg/L) for the years 2019 to 2022 (a - d) compared to the suspended matter concentrations (mg/L) from the Moselle.

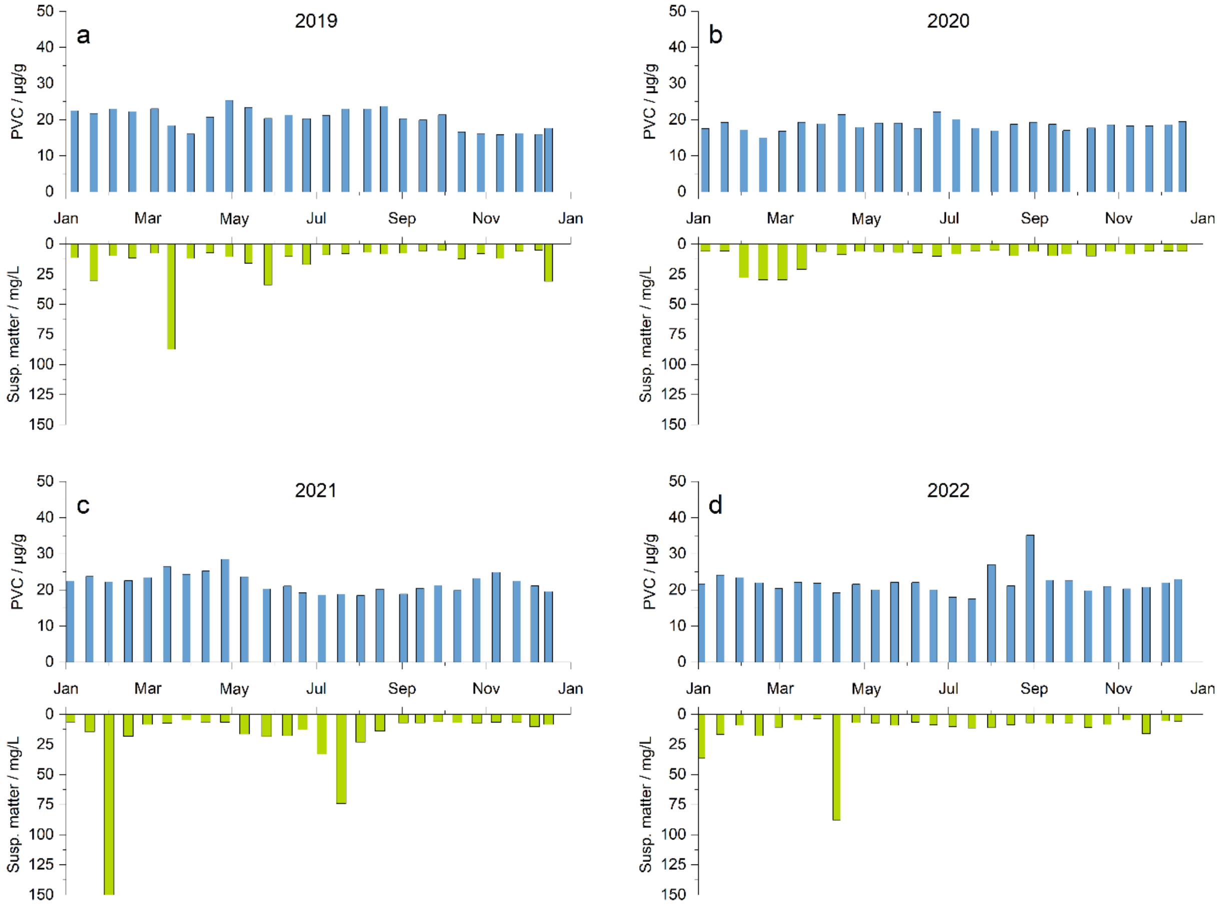

Figure S.4: Comparison of the behavior of the PVC concentration (mg/g) over the years 2019 to 2022 (a – d) in the collected suspended matter samples and the suspended matter concentration (mg/L) in the Rhine.

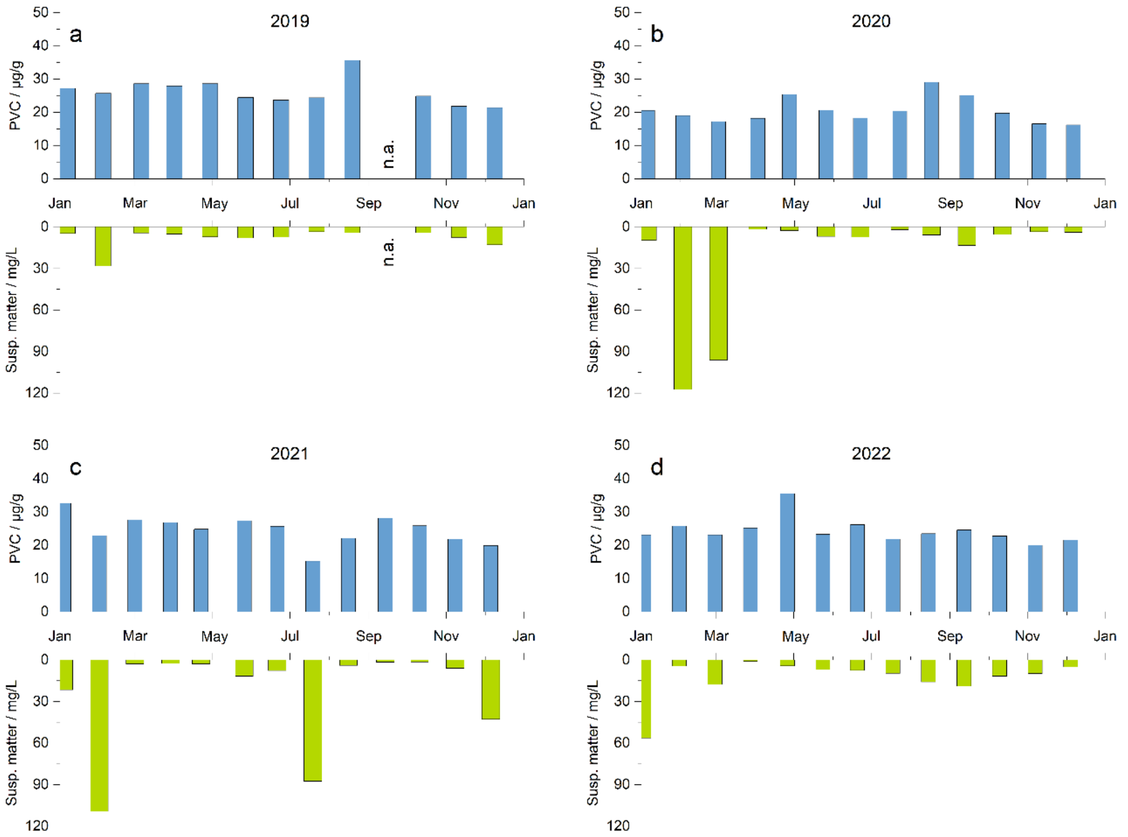

Figure S.5: Comparison of the behavior of the PVC concentration (mg/g) in the Moselle over the years 2019 to 2022 (a – d) in the collected suspended matter samples and the suspended matter concentration (mg/L). October 2019 was not applicable (n.a.).

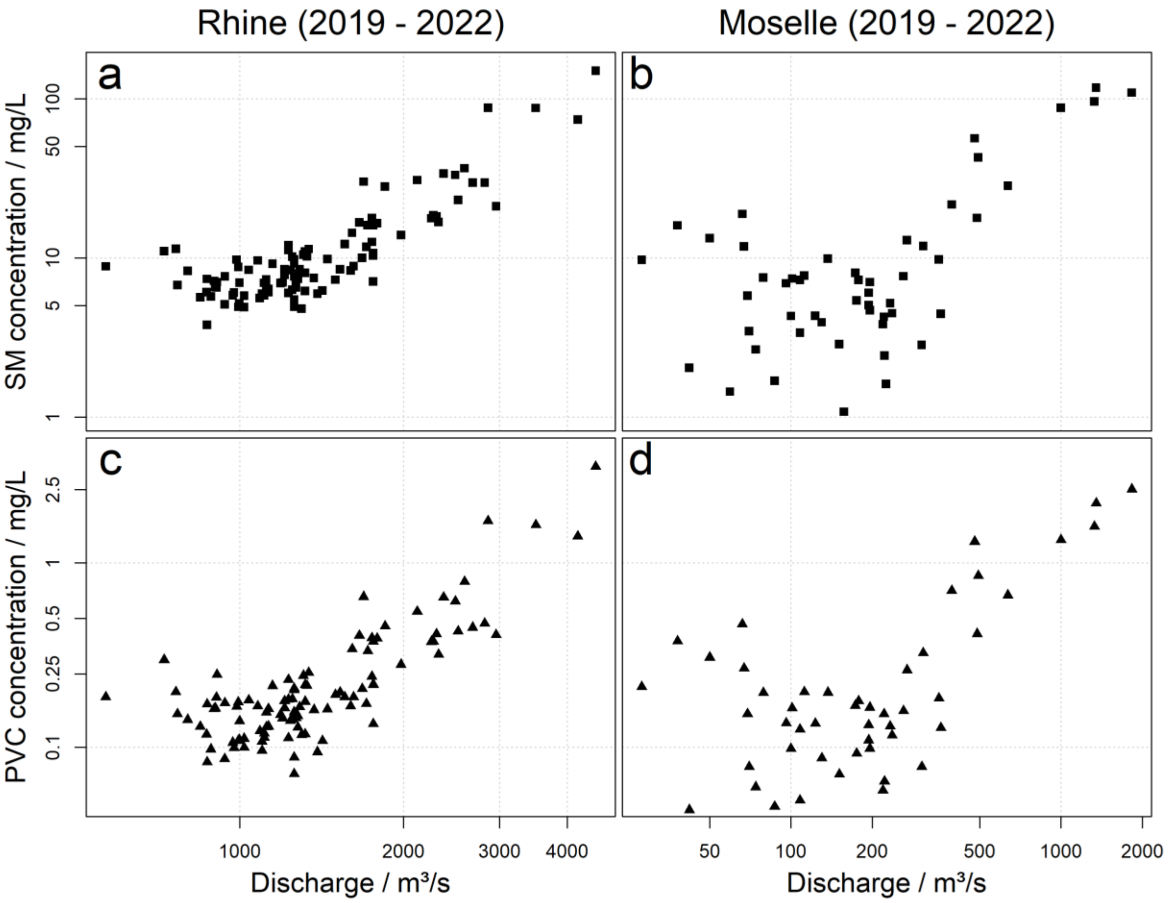

Figure S.6: Logarithmic scale depiction of the SM concentration (mg/L; a) Rhine, b) Moselle) and the PVC concentration (µg/L; c) Rhine, d) Moselle) against the discharge (m³/s) of the respective sampling day.

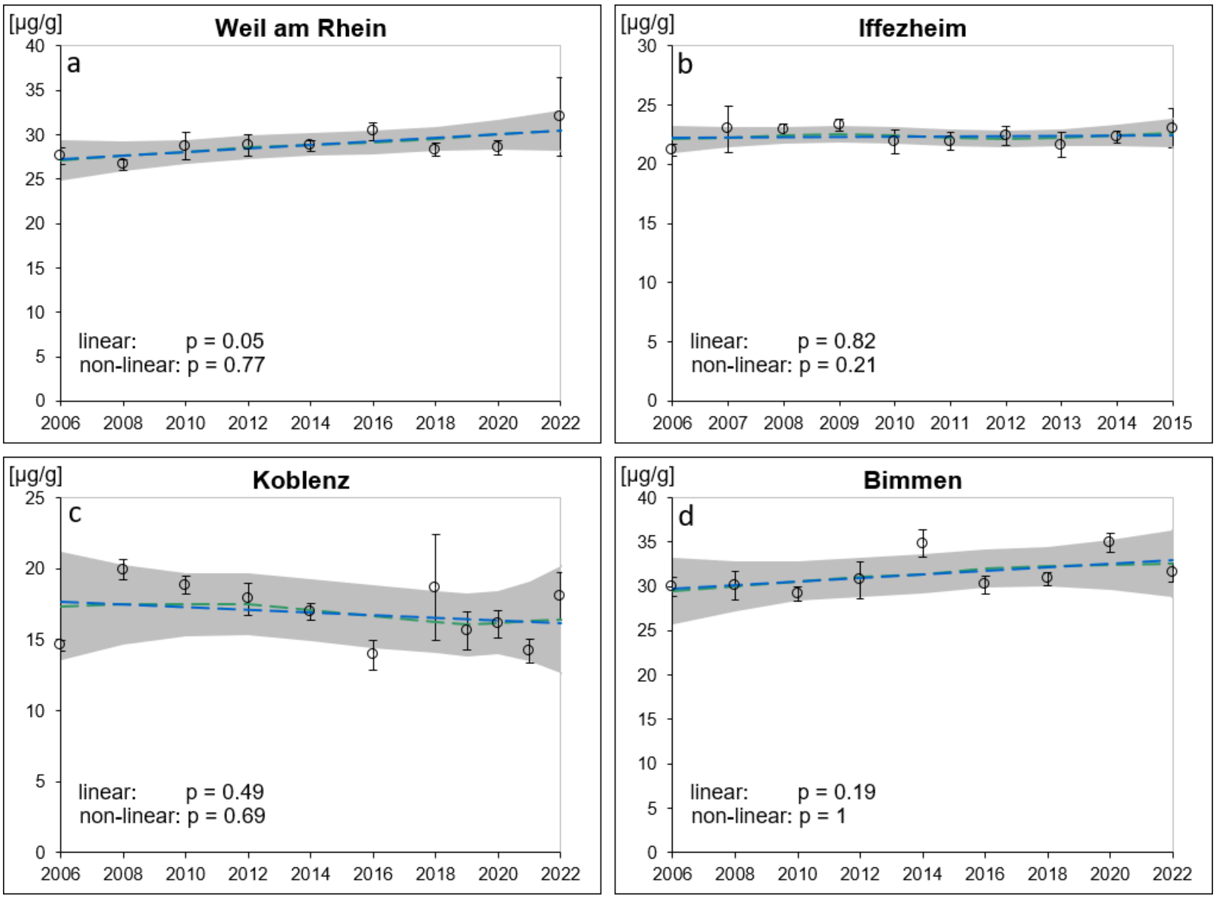

Figure S.7: LOESS trend analysis of the PVC concentrations (µg/g) at Weil (a), Iffezheim (b), Koblenz (c) and Bimmen (d) from 2006 to 2022. Blue dashed line: linear regression. Green dashed line: LOESS trend calculation. Grey area: error band of the 95% confidence interval.

References

(1) Dierkes, G.; Lauschke, T.; Becher, S.; Schumacher, H.; Foldi, C.; Ternes, T. Quantification of microplastics in environmental samples via pressurized liquid extraction and pyrolysis-gas chromatography. *Anal Bioanal Chem* **2019**, *411* (26), 6959-6968. DOI: 10.1007/s00216-019-02066-9.

(2) Kamp, J.; Dierkes, G.; Schweyen, P. N.; Wick, A.; Ternes, T. A. Quantification of Poly(vinyl chloride) Microplastics via Pressurized Liquid Extraction and Combustion Ion Chromatography. *Environmental Science & Technology* **2023**, *57* (12), 4806-4812. DOI: 10.1021/acs.est.2c06555.

(3) Boehlich, M. J.; Strotmann, T. The Elbe Estuary. *Die Küste* **2008**, (74), 288-306.

(4) Vinther, N.; Christiansen, C.; Bartholdy, J.; Sørensen, C.; Lund-Hansen, L. C. Sediment transport across a tidal divide in the Danish Wadden Sea. *Geografisk Tidsskrift-Danish Journal of Geography* **2004**, *104* (1), 71-86. DOI: 10.1080/00167223.2004.10649505.

(5) Gebhardt, M.; Gerstner, N.; Thorenz, C. The Impact of Overflow and Underflow Gates on the Sediment Transport in Impounded Rivers. *Wasserwirtschaft* **2013**, *Jg. 103* (11/2013), 16-22.

(6) Titow, W. V. *PVC plastics: properties, processing, and applications*; Springer Science & Business Media, 2012.

(7) Nord, G.; Esteves, Michel; Lapetite, J.-M.; Hauet, A. Effect of particle density and inflow concentration of suspended sediment on bedload transport in rill flow. **2009**, *34* (2), 253-263. DOI: https://doi.org/10.1002/esp.1710.

(8) Naden, P. S. The Fine-Sediment Cascade. In *Sediment Cascades*, 2010; pp 271-305.

(9) Watkins, L.; McGrattan, S.; Sullivan, P. J.; Walter, M. T. The effect of dams on river transport of microplastic pollution. *Sci Total Environ* **2019**, *664*, 834-840. DOI: 10.1016/j.scitotenv.2019.02.028.
